# The Association between Training Frequency, Symptoms of Overtraining and Injuries in Young Men Soccer Players

**DOI:** 10.3390/ijerph20085466

**Published:** 2023-04-11

**Authors:** Filipe Rodrigues, Diogo Monteiro, Ricardo Ferraz, Luís Branquinho, Pedro Forte

**Affiliations:** 1 ESECS-Polytechnic of Leiria, 2411-901 Leiria, Portugal; 2 Life Quality Research Center, 2040-413 Leiria, Portugal; 3 Research Centre in Sports Sciences, Health Sciences, and Human Development, 6201-001 Covilhã, Portugal; 4 Department of Sports, University of Beira Interior, 6201-001 Covilhã, Portugal; 5 Sport Department, Higher Institute of Educational Sciences of the Douro, 4560-708 Penafiel, Portugal; 6 CI-ISCE, ISCE Douro, 4560-708 Penafiel, Portugal; 7 Department of Sports, Instituto Politécnico de Bragança, 5300-253 Bragança, Portugal

**Keywords:** sports, pedagogy, injury risk, physical health, overtraining

## Abstract

Overtraining is a prevalent issue among young men soccer players, particularly those who are driven to enhance their skills. While an intense training volume and effort might contribute to athletic growth, it can also have negative implications, including injury. The current study aimed at examining the association between training frequency, symptoms of overtraining and injuries in young men soccer players. A path analysis approach was used to examine the causal relationships between variables. The sample consisted of 189 young men soccer players aged 13–17 years old (age = 14.81, SD = 1.37). Participants reported that they were training, on average, 5.77 days (SD = 1.53) per week. Athletes were competing at a regional (n = 100) or national (n = 89) level. Concerning injuries, participants indicated, on average, 2.03 (SD = 1.16) injuries since they started practicing soccer. The results displayed a significant association, as theoretically expected, namely: (i) training frequency was significantly associated with overtraining symptoms (β = 0.15 [IC95% = 0.01, 0.29]); (ii) overtraining symptoms were significantly associated with the number of injuries (β = 0.19 [IC95% = 0.02, 0.35]). An indirect effect between training frequency and injuries was also observed (β = 0.15 [IC95% = 0.01, 0.29]). Thus, there is preliminary evidence that overtraining symptoms could play a mediating role. In conclusion, investigating the links between overtraining symptoms and injury in young men soccer players is critical, as it can assist in identifying overtraining warning signs, promote young players’ health and safety, customize training regimens to individual needs, and contribute to a better understanding of sports-related injuries.

## 1. Introduction

Soccer is a physically demanding sport in which players must participate in high-intensity actions, such as running, jumping, and tackling, often for prolonged periods [1,2]. Athletes must go through intense training regimens meant to improve their strength, endurance, and agility in order to compete at their best. Overtraining can develop if the intensity, frequency, and length of training surpass the ability of the body to recover [3]. Overtraining syndrome is a prevalent issue among soccer players, and it can cause a variety of physical and mental symptoms that can have an effect on their performance as well as their overall health and well-being [4]. The characterization of overtraining syndrome is an important topic for researchers, coaches, and athletes. Understanding the mechanisms underlying overtraining and its impact on injuries might aid in identifying possible risk factors and developing effective preventative and management techniques. Furthermore, recognizing the signs of overtraining and treating it early can assist in reducing the negative impacts of the condition and promote optimal performance and well-being in soccer players [5].

Overreaching is a short-term phenomenon that occurs when athletes engage in intense training over a short period of time, such as during a pre-competition phase [6]. It is a planned and regulated type of training that is intended to produce temporary exhaustion, but with proper rest and recovery, performance returns to normal or even improves. Overreaching is regarded as a normal and necessary component of sports training that can result in improved performance [7]. Overtraining has been defined in a variety of ways, but one widely accepted definition is prolonged maladaptation to high levels of exercise stress that results in decreased performance and mood state, with symptoms that can continue for weeks or months [8]. Some of the objective symptoms of overtraining syndrome include decreased performance, elevated heart rate, elevated cortisol levels, decreased muscle strength and power, increased perceived exertion, sleep disturbances, and reduced immune function [7]. The rationale behind this description is that overtraining is more than just training too much. It is a complicated physiological and psychological response to stress that results in chronic exhaustion, decreased performance, and negative mood states. Athletes who undergo prolonged and severe emotional and physical tiredness may experience burnout [9,10]. It is defined as a loss of interest, motivation, and enjoyment in athletics, and it can result in a variety of psychological and physical symptoms, including sadness, anxiety, sleep difficulties, and impaired performance. Burnout is frequently accompanied by high levels of stress, a sense of loss of control, and mental and physical tiredness [11]. The transition from overreaching to overtraining to burnout is a complex phenomenon that involves a gradual shift from a normal and adaptive response to training to a maladaptive state of physical and psychological fatigue, with the negative effects on performance, health, and well-being becoming more severe and lasting [12].

Injuries are characterized by localized discomfort, edema, and a decreased range of motion. They can be acute or chronic, and the cause can be a single traumatic incident or a series of microtraumas [13]. Physical injuries can include muscle strains, ligament sprains, fractures, and head injuries such as concussions. Additionally, soccer players are at risk of overuse injuries, such as tendinitis or stress fractures due to the repetitive nature of certain movements during play. In soccer athletes, overtraining symptoms have been demonstrated to be associated with injury risk. Systematic research of male and female soccer athletes discovered that those who reported more fatigue, muscle discomfort, and lower performance were more likely to be injured [14]. Similarly, another study discovered that players who reported more than three overtraining symptoms, such as weariness, sleep disruptions, and mood abnormalities, were more likely to get injured [8]. However, there is scarcity of research assessing overtraining symptoms and injuries in young soccer athletes [14,15]. First, examining the relationships between training frequency, overtraining symptoms, and injuries in young men soccer players can aid in identifying risk factors for injury. Second, investigating the links between training frequency, overtraining symptoms, and injuries in young men soccer players can aid in the prevention of overtraining syndrome. Overtraining syndrome is a potentially fatal illness that can result in physical and emotional exhaustion, poor sports performance, and burnout [16]. Finally, investigating the relationships between training frequency, overtraining symptoms, and injuries in young men soccer players can aid in improving athletic performance. When athletes are overtrained, their sports performance may decrease as a result of physical and mental exhaustion.

### Novelty of the Current Study

Studying the association between training frequency, symptoms of overtraining, and injuries in Portuguese young men soccer players can contribute to the scientific knowledge on the topic and potentially provide new insights. First, to the best of our knowledge, this study is the first to examine these specific associations in Portuguese young men soccer players and thus could provide novel insights into the prevalence and risk factors of overtraining and injuries in this population. Portugal has a strong football culture and has produced many successful football players and teams over the last few years. As a result, there is a significantly increasing amount of research on football in Portugal. Some of the areas of research related to football in Portugal include sports medicine, sports psychology, training methods, and performance analysis. Considering the need to understand the variables that produce top players and teams, analyzing the associations between performance-related indicators and sports volume seems of the utmost importance. This study uses contemporary statistical tests, namely path analysis, to explore the associations among variables, since previous studies have conducted traditional correlational analyses. Path analysis is a statistical technique that allows researchers to test complex causal models by examining the relationships between variables in a directed graph, considering both direct and indirect effects by accounting for the variance in multiple variables simultaneously. In contrast to correlational analysis, which only examines the strength and direction of the association between variables, path analysis allows researchers to test the direction and magnitude of causal effects among variables, and to estimate the indirect effects of variables on outcomes through intervening variables. Finally, understanding the relationship between training frequency and overtraining symptoms can help coaches and trainers design programs that prioritize athlete health and wellbeing.

The current study aimed at examining the association between training frequency, symptoms of overtraining and injuries in young men soccer players. We used a path analysis approach to examine the causal relationships between variables [17]. This method can help researchers to better understand the mechanisms underlying the relationships between training frequency, overtraining symptoms, and reported injuries in a systematic and efficient manner. We hypothesized that training frequency, as well as experience, would positively relate to overtraining symptoms [15]. Consequently, overtraining symptoms would positively associate with the risk of injury [8].

## 2. Materials and Methods

### 2.1. Participants

Regarding the parameters to be estimated (i.e., 4 parameters), Hair et al. [17] recommend a 10:1 ratio (participants per parameter to be estimated). Thus, the minimum sample size required for statistical power should be approximately 40 participants. To account for potential missing data (10%), we considered 44 participants as the minimum sample size. For additional statistical power calculations, the Soper [18] a priori sampling calculator for factor analysis was used to calculate the minimum sample size required for this study to be valid and reliable. The following inputs were used: anticipated effect size = 0.15 (small effect); desired statistical power = 0.95; the number of observed variables = 3; probability level = 0.05. The results suggest that the minimum number of participants is 143 for the results to be valid and reliable. In the present study, the sample consisted of 189 participants aged 13–17 years old (age = 14.81, SD = 1.37). Participants reported that they were training per week on average for 5.77 days (SD = 1.53). Athletes were competing at the regional (n = 100) or national (n = 89) level. For inclusion, we considered those who met the following inclusion criteria: (i) aged between 13 and 17 years; (ii) provide informed consent from parents or legal guardians to participate and also from themselves; and (iii) be an active athlete amateur or professional.

### 2.2. Procedures

Data collection procedures were carried out in compliance with the Helsinki Declaration and its subsequent revisions, or comparable ethical norms [19]. Prior to data collection, the Ethics Committee of the Scientific Board of the Higher Institute of Educational Sciences of the Douro (reference number: 31142) approved all procedures carried out during the study. Then, considering the convenience sampling method, soccer clubs were contacted, the research objectives were presented, and, subsequently, consent was granted for the data collection procedures. Coaches were contacted before training sessions and researchers explained the objective of the study. Subsequently, the researchers also explained the purpose of the study to parents and legal guardians. It was fully explained that the data would be used only for research purposes and anonymously, that participation was voluntary, and that any individual could withdraw from the study at any time even after starting it. After obtaining the signed written informed consent, the athletes were contacted and the objective of the study was explained to them, as well as information related to the foundation of the research project. In addition, 2 other questionnaires were applied (i.e., sociodemographic questionnaire and the overtraining questionnaire from the Société Françoise de Medicine du Sport) and each of them took approximately 10 min to complete. The athletes did not receive a reward, but their efforts were recognized and thanked.

### 2.3. Instruments

Participants completed a sociodemographic questionnaire measuring age, experience, week training frequency, and how many injuries did they report since practicing formal soccer. The researchers provided examples of injuries for clarity. Participants also completed the overtraining questionnaire of the Société Françoise de Medicine du Sport Portuguese version [20]. This measure comprises 52 items (e.g., “*My performance level has deteriorated*”) and responses are provided on a Likert-type scale ranging from 0 (never) and 3 (always).

### 2.4. Statistical Analysis

Using the IBM SPSS STATISTICS version 25.0 software, descriptive statistics such as means and standard deviations, as well as bivariate correlations between all variables under consideration, were generated. To determine the statistical significance of departures from the normal distribution, the Kolmogorov–Smirnov test was used. For the referred analyses, a significance value ≤ 0.05 was assumed to reject the null hypothesis.

Afterwards, a path analysis was conducted using a two-step approach. First, a confirmatory factor analysis was performed, followed by a structural equation model considering all variables under analysis was performed. These analyses were carried out with the robust maximum likelihood estimator from Mplus, version 7.4. Direct and indirect effects were analyzed according to standardized coefficients and their respective 95% Confidence Interval (CI95%). Regression paths were considered significant if the CI95% did not include zero.

## 3. Results

The mean, standard deviation, and correlations between study variables are reported in Table 1. The Kolmogorov–Smirnov test indicates no deviation of normality in all variables. The results indicated several significant associations as theoretically expected, namely: (i) training frequency was significantly associated with overtraining symptoms; (ii) overtraining symptoms were significantly associated with a number of injuries; (iii) training experience was negatively associated with overtraining symptoms but positively with a number of injuries. Concerning injuries, participants indicated on average 2.03 (SD = 1.16) injuries since they started practicing sports.

**Figure 1:**
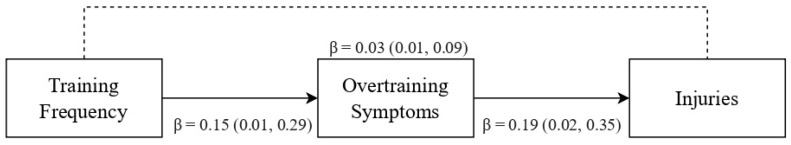
Path model. Notes: β = standardized beta coefficients; straight lines = direct regression path; dotted lines = indirect regression path; within brackets = 95 percent confidence interval.

Figure 1 displays the proposed path model. Positive and significant associations were observed among variables. The result displays an indirect effect between training frequency and injuries. Thus, there is preliminary evidence that overtraining symptoms could play a mediating role.

## 4. Discussion

The present study examined the association between training frequency, symptoms of overtraining, and injuries in young men soccer players. This analysis was conducted according to previously proposed assumptions [14,15]. Examining correlations, positive and significant associations were observed among training frequency, symptoms of overtraining, and injuries. These results have been theoretically and systematically supported in another sport context [3] and, specifically, in soccer athletes [14,15].

Examining the proposed model, the current research adds new insights into how the causal relationship works between training frequency, symptoms of overtraining, and injuries. Starting with the association between training frequency and overtraining symptoms, there is evidence that a greater weekly frequency could lead to the perception of greater symptoms of overtraining. The present results support previous studies [15,21], suggesting that athletes that participate in high-frequency training regimes are more prone to suffer from overtraining symptoms such as fatigue, injury, and diminished performance. This is also the case for young soccer athletes, since current results show that frequency significantly predicts overtraining symptoms.

Overtraining symptoms predicted injuries in young men soccer players. This result is in accordance with previous studies [22,23], indicating that fatigue, irritability, poor performance, loss of appetite, and increased susceptibility to illness can lead to more serious problems, such as muscle strains, joint pain, stress fractures, and even mental health issues such as depression and anxiety [3,24,25]. It is critical to understand the unique challenges that young men soccer players encounter. Many of these athletes are still growing and developing their physical and mental skills, making them more susceptible to injury. Furthermore, they frequently have schedules that make balancing training, school, and other commitments more demanding [26]. The present results also showed an indirect effect between training frequency and injuries, possibly mediated by overtraining symptoms. Young men soccer players who train more frequently are at a higher risk of injury.

### 4.1. Practical Implications

On account of the wide range of physical demands placed on soccer players in various moments (i.e., training sessions and competitive games), indications and symptoms of overtraining specific to different forms of exercise may be widespread [27]. While physical training demands were not measured, excessive resistance training volumes and intensities have been proven to create distinct fatigue profiles in overreaching athletes when compared to endurance training [28]. Additionally, the sensitivity to exhaustion of different physical attributes (for example, strength vs. power) varies. As a result, the onset of overtraining may occur at different times depending on the athlete and the physical performance test assessed. Furthermore, while the cause of overtraining is widely considered to be multidimensional, it is possible that athletes that incur different demands also experience different symptoms. As a result, researching overtraining symptoms as a homogeneous outcome that affects all athletes in the same way and attempting to quantify the symptoms of athletes from a variety of athletic endeavors is likely oversimplified. Thus, more studies are warranted, since there are various variables that can contribute to the correct reporting of overtraining in order to appropriately monitor and quantify the signs and associated symptoms. First, a baseline in a valid and reliable test that examines a relevant physical characteristic must be established. This is essential to confirm that a performance change has occurred. Second, regular testing is required to see if performance has degraded. Yet, if an athlete has reached a condition of overtraining, physically assessing the athlete during this period may be unfavorable and exacerbate the fatigue and psychological burden of the athlete. Third, it must be clearly proven that a decline in performance is not an immediate reaction to past sessions of training. According to research on tapering and peaking, performance super-compensation can take up to four weeks after reducing training volume, and the timing of super-compensation can fluctuate between athletes despite comparable training loads [29].

The results from the current study have theoretical and empirical implications. We can contribute to a better knowledge of sports-related injuries and their prevention by investigating the links between overtraining and injury in young men soccer players by considering exercise frequency. This research can assist inform the creation of injury prevention programs and improve the safety of young athletes and their ability levels in soccer. Coaches can assist young men soccer players realize their full potential while decreasing the risk of injury by monitoring overtraining signs and changing training accordingly. This is especially significant given soccer’s competitive character and the pressure that young sportsmen may feel to achieve at a high level. In addition, by investigating the links between these symptoms and injury in young men soccer players, we can gain a better understanding of the warning indications of overtraining and implement preventative measures before an injury develops.

Coaches can monitor the training load and modify training plans as needed. Coaches can determine when a young soccer athlete is at risk of overtraining and adapt the training program to prevent further symptoms by tracking the volume, intensity, and frequency of training. To limit the risk of injury and overtraining, coaches can reduce the volume or intensity of training, increase rest periods, or change training activities. Prioritizing recovery exercises is another important method for coaches to avoid overtraining symptoms. This includes methods such as optimal diet, hydration, and rest and recovery intervals. Coaches can also add recovery exercises into their training regimens. Coaches can impact the psychological components of training in addition to the physical parts of the training load. Overtraining syndrome is not only a physical condition, but it can also harm an athlete’s emotional health. Athletes can benefit from coaches creating a positive team culture that fosters mental health and well-being. Team-building exercises, open communication lines, and establishing a positive team environment are examples of such activities.

### 4.2. Limitations and Research Agenda

A limitation of the present study that should be acknowledged is that we measured overtraining symptoms at an initial assessment. It would be interesting in future studies to explore the extent to which self-reported and other measures of fatigue-related symptoms (e.g., training load, blood samples) can predict injuries. In addition, athletes self-reported their previous injuries and, thus, we are unable to ascertain the validity of responses. Future studies should assess injuries with the medical staff of each player and also categorize injuries by the degree of severity. Likewise, assessing overtraining symptoms over a longer period of time (e.g., 1 year) could be of interest in measuring the impact of motivational and emotional determinants beyond behavior factors (i.e., frequency and intensity). Future studies should also test our hypothesized model in adult athletes and in different cultures to test for the generalizability of the current results.

## 5. Conclusions

The current study supports prior studies that found a link between training frequency, overtraining symptoms, and injuries in young men soccer players. The study emphasizes the need of monitoring young men soccer players’ training frequency and intensity to reduce the risk of overtraining and injury. Coaches and trainers should be aware of the indications of overtraining and take appropriate precautions to avoid and manage the negative consequences of overtraining. Young men soccer players can limit their risk of overtraining and injury by taking these precautions, ensuring their long-term health and athletic success.

## Figures and Tables

**Table 1:** Descriptive statistics and correlations between study variables.

Variable	M	SD	1	2	3	4	5
1. Age	14.81	1.37	1	0.65 **	0.26 **	−0.12	0.25 **
2. Training experience	7.88	1.78	0.63 **	1	0.02	−0.43	0.13
3. Frequency	5.77	1.15	0.30 **	0.04	1	0.12	0.07
4. Overtraining symptoms	0.73	0.29	−0.01	−0.39 **	0.15 *	1	0.15 *
5. Injury	2.03	1.16	0.32 *	0.15 *	0.10	0.19 **	1

**Notes:** M = Mean; SD = Standard-Deviation; below diagonal line = bivariate correlations; above diagonal line = partial correlations controlling for competitive level; * = *p* < 0.05; ** = *p* < 0.01.

## Data Availability

Data are available upon reasonable request to the corresponding author.

## References

[1] Branquinho L., Ferraz R., Marques M.C. (2021). 5-a-Side Game as a Tool for the Coach in Soccer Training. Strength Cond. J..

[2] Teixeira J.E., Forte P., Ferraz R., Leal M., Ribeiro J., Silva A.J., Barbosa T.M., Monteiro A.M. (2021). Monitoring Accumulated Training and Match Load in Football: A Systematic Review. Int. J. Environ. Res. Public Health.

[3] Kreher J.B., Schwartz J.B. (2012). Overtraining Syndrome: A Practical Guide. Sports Health.

[4] Djaoui L., Haddad M., Chamari K., Dellal A. (2017). Monitoring Training Load and Fatigue in Soccer Players with Physiological Markers. Physiol. Behav..

[5] Nobari H., Fani M., Pardos-Mainer E., Pérez-Gómez J. (2021). Fluctuations in Well-Being Based on Position in Elite Young Soccer Players during a Full Season. Healthcare.

[6] Nobari H., Fani M., Clemente F.M., Carlos-Vivas J., Pérez-Gómez J., Ardigò L.P. (2021). Intra- and Inter-Week Variations of Well-Being Across a Season: A Cohort Study in Elite Youth Male Soccer Players. Front. Psychol..

[7] Halson S.L., Jeukendrup A.E. (2004). Does Overtraining Exist? An Analysis of Overreaching and Overtraining Research. Sport. Med..

[8] Meeusen R., Duclos M., Foster C., Fry A., Gleeson M., Nieman D., Raglin J., Rietjens G., Steinacker J., Urhausen A. (2013). Prevention, Diagnosis, and Treatment of the Overtraining Syndrome: Joint Consensus Statement of the European College of Sport Science and the American College of Sports Medicine. Med. Sci. Sports Exerc..

[9] Liu M., Zhao X., Liu Z. (2022). Relationship between Psychological Distress, Basic Psychological Needs, Anxiety, Mental Pressure, and Athletic Burnout of Chinese College Football Athletes during the COVID-19 Pandemic. Sustainability.

[10] Sarmento H., Frontini R., Marques A., Peralta M., Ordoñez-Saavedra N., Duarte J.P., Figueiredo A., Campos M.J., Clemente F.M. (2021). Depressive Symptoms and Burnout in Football Players: A Systematic Review. Brain Sci..

[11] Groenewal P.H., Putrino D., Norman M.R. (2021). Burnout and Motivation in Sport. Psychiatr. Clin. North Am..

[12] Wilczyńska D., Qi W., Jaenes J.C., Alarcón D., Arenilla M.J., Lipowski M. (2022). Burnout and Mental Interventions among Youth Athletes: A Systematic Review and Meta-Analysis of the Studies. Int. J. Environ. Res. Public Health.

[13] López-Valenciano A., Ruiz-Pérez I., Garcia-Gómez A., Vera-Garcia F.J., De Ste Croix M., Myer G.D., Ayala F. (2020). Epidemiology of Injuries in Professional Football: A Systematic Review and Meta-Analysis. Br. J. Sports Med..

[14] McCall A., Carling C., Davison M., Nedelec M., Le Gall F., Berthoin S., Dupont G. (2015). Injury Risk Factors, Screening Tests and Preventative Strategies: A Systematic Review of the Evidence That Underpins the Perceptions and Practices of 44 Football (Soccer) Teams from Various Premier Leagues. Br. J. Sports Med..

[15] Carvalho R., Monteiro D., Rodrigues F. (2022). Relación Entre Estados de Ánimo y Síndrome de Overtraining En Jóvenes Deportistas. Cuad. Psicol. del Deport..

[16] Granz H.L., Schnell A., Mayer J., Thiel A. (2019). Risk Profiles for Athlete Burnout in Adolescent Elite Athletes: A Classification Analysis. Psychol. Sport Exerc..

[17] Hair J.F., Babin B.J., Anderson R.E., Black W.C. (2019). Multivariate Data Analysis.

[18] Soper D. (2022). A-Priori Sample Size Calculator for Structural Equation Models [Software], Version 4.0. https://www.danielsoper.com/statcalc.

[19] Association W.M. (2013). World Medical Association Declaration of Helsinki: Ethical Principles for Medical Research Involving Human Subjects. JAMA.

[20] Fernandes J., Nogueira R., Andrade F., Freitas D.S., Bara Filho M. (1986). Tradução E Adaptação Do Questionário De Sintomas Clínicos Do Overtraining. Coleção Pesqui. Educ. Física.

[21] Cadegiani F.A., Kater C.E. (2019). Novel Insights of Overtraining Syndrome Discovered from the EROS Study. BMJ Open Sport Exerc. Med..

[22] Mandorino M., Figueiredo A.J., Cima G., Tessitore A. (2021). A Data Mining Approach to Predict Non-Contact Injuries in Young Soccer Players. Int. J. Comput. Sci. Sport.

[23] Mandorino M., Figueiredo A.J., Condello G., Tessitore A. (2022). The Influence of Maturity on Recovery and Perceived Exertion, and Its Relationship with Illnesses and Non-Contact Injuries in Young Soccer Players. Biol. Sport.

[24] Kibler W.B., Chandler T.J., Stracener E.S. (1992). Musculoskeletal Adaptations and Injuries Due to Overtraining. Exerc. Sport Sci. Rev..

[25] Pfirrmann D., Herbst M., Ingelfinger P., Simon P., Tug S. (2016). Analysis of Injury Incidences in Male Professional Adult and Elite Youth Soccer Players: A Systematic Review. J. Athl. Train..

[26] Jayanthi N., Pinkham C., Dugas L., Patrick B., LaBella C. (2013). Sports Specialization in Young Athletes: Evidence-Based Recommendations. Sports Health.

[27] Carrard J., Rigort A.C., Appenzeller-Herzog C., Colledge F., Königstein K., Hinrichs T., Schmidt-Trucksäss A. (2022). Diagnosing Overtraining Syndrome: A Scoping Review. Sports Health.

[28] Fry A.C., Kraemer W.J. (1997). Resistance Exercise Overtraining and Overreaching: Neuroendocrine Responses. Sport. Med..

[29] Morin J.B., Capelo-Ramirez F., Rodriguez-Pérez M.A., Cross M.R., Jimenez-Reyes P. (2022). Individual Adaptation Kinetics Following Heavy Resisted Sprint Training. J. Strength Cond. Res..

